# Ginsenoside Rg2 from *Panax* spp.: Diverse Biological Activities, Mechanisms and Prospect

**DOI:** 10.3390/molecules31183297

**Published:** 2026-09-17

**Authors:** Jiahui Lu, Ruomeng Li, Rui Liu, Lanqi Liu, Biyu Lan, Zehua Wang, Guizhen Wang

**Affiliations:** College of Biological and Food Engineering, Jilin Engineering Normal University, Changchun 130052, China; lujiahui@stu.jlenu.edu.cn (J.L.); liruomeng@stu.jlenu.edu.cn (R.L.); liurui@stu.jlenu.edu.cn (R.L.); liulanqi@stu.jlenu.edu.cn (L.L.); lanbiyu@stu.jlenu.edu.cn (B.L.); wangzehua@stu.jlenu.edu.cn (Z.W.)

**Keywords:** ginsenoside Rg2, 20(S)/20(R)-epimer, pharmacological activity, molecular mechanism, signal pathway, cytoprotective effect

## Abstract

As a well-known medicinal and edible traditional Chinese medicine, ginseng is rich in diverse active ingredients and exerts broad pharmacological effects. Among them, ginsenoside Rg2 (Rg2) has become a research hotspot due to its notable biological activities. Rg2 exhibits multiple pharmacological activities, including myocardial protection, nervous system regulation, and anti-inflammation, anti-tumor, and anti-oxidative stress effects, by regulating multiple signaling pathways such as AKT, MAPK-ERK, and NF-κB/NLRP3. It also shows favorable biological functions in protecting against pregnancy-related brain injury, intervening in osteoporosis, delaying muscle atrophy, regulating metabolism, and modulating immunity, with preliminary oral safety profiles reported in rodent studies. This paper provides a narrative overview of the research progress on the pharmacological effects and mechanisms of Rg2, with the aim of offering a theoretical foundation for the development of natural drugs and the utilization of ginseng, as well as a reference for clinical trials and combination therapy strategies.

## 1. Introduction

Ginseng, the dried root and rhizome of *Panax species* (Araliaceae), is native to China and the Korean Peninsula, and is a globally valued medicinal herb [1]. It has been traditionally recorded as a superior herb in *Shennong Bencao Jing* [2], and as a remedy for all deficiencies in the Compendium of Materia Medica [3]. Modern research and WHO recognition support its anti-fatigue and stress-resistant properties [4,5], indicating broad clinical potential [6,7].

Ginseng is composed of ginsenosides, ginseng polysaccharides, and amino acids, among which triterpenoid ginsenosides serve as the core bioactive components. These compounds generally appear as white amorphous powders or colorless crystals, with a slightly sweet–bitter taste, good water solubility, and poor solubility in lipophilic organic solvents. According to the structural characteristics of aglycones, ginsenosides are categorized into three major types, among which dammarane-type tetracyclic triterpenoid saponins are the most widely reported [8]. Ginsenoside Rg2 is a typical dammarane-type saponin that naturally exists as two epimers, 20(S)-ginsenoside Rg2 [20(S)-Rg2] and 20(R)-ginsenoside Rg2 [20(R)-Rg2]. Both epimers share the same molecular formula C_42_H_72_O_13_ with a molecular weight of 785.0. The chemical structures of these two Rg2 isoforms are displayed in Table 1 [9]. Benefiting from diverse active constituents, ginseng exhibits multiple pharmacological activities, including anti-inflammatory, anti-fatigue, antioxidant, and anti-tumor effects [10]. Ginsenoside Rg2 possesses typical functional groups that constitute the structural basis for its biological performance. It contains abundant hydroxyl groups distributed on the aglycone skeleton and terminal sugar moieties, a C24-C25 unsaturated double bond on the side chain, and an O-glycosidic bond that links the disaccharide unit at the C6 position. These structural characteristics determine the polarity and water solubility of Rg2, and provide potential binding sites for interactions with biological targets. Moreover, subtle differences in hydroxyl substitution patterns and C20 chiral configurations are critical factors driving the distinct biological activities of Rg2 epimers. Indeed, 20(S)- and 20(R)-Rg2 have been reported to exhibit stereospecific differences in both pharmacodynamic and pharmacokinetic profiles. [11,12].

Previous reviews have mainly focused on nervous-system effects of Rg2 and Rh1 [13]; however, few publications have comprehensively collated its full-spectrum biological functions, multi-target mechanisms, and translational research prospects. Notably, 20(S)- and 20(R)-Rg2 exhibit significantly different biological potency, whereas most original studies fail to specify the epimer type used in experiments, resulting in inconsistent and incomparable research conclusions. Accordingly, this review attempts to systematically document the epimer information of Rg2 as reported in original research, aiming to provide a useful reference for subsequent translational research on ginsenoside Rg2.

## 2. Methods

Literature searches were performed in PubMed and Web of Science from database inception to June 2026. Search string for PubMed: (ginsenoside Rg2 [Title/Abstract]) AND (epimer OR stereoisomerism OR “structure-activity relationship”); search string for Web of Science: TS = (“ginsenoside Rg2”) AND TS = (epimer OR stereoisomerism OR “structure-activity relationship”).

The initial retrieval yielded 128 records, and 81 references were finally cited after deduplication and screening. Of these, 30 were original experimental articles reporting pharmacological data of Rg2, which are summarized in Table 2 and Table 3; the remaining references (reviews, background literature, and one plotting-software citation) were cited for contextual purposes only. Experimental details extracted from the 30 primary studies include animal or cell models, epimer subtype, dosage, administration route, treatment duration, outcome direction, and positive control data. As a narrative review, this manuscript did not strictly follow the PRISMA workflow, and representative studies reflecting major research progress were prioritized.

## 3. Preparation, Pharmacokinetics and Toxicology

### 3.1. Preparation of Ginsenoside Rg2

Ginsenoside Rg2 can be efficiently prepared via biotransformation using microbial enzymes. A simple and efficient transformation system using *Cellulosimicrobium* sp. 21 was developed in a phosphate buffer system, achieving a biotransformation efficiency of 89.8% (molar ratio) in a 2 L reaction system [14]. Alternatively, recombinant β-glucosidase (BglPC28) from *Pseudonocardia* sp. Gsoil 1536 can effectively convert ginsenoside Re to Rg2(S) with high specificity and yield [15]. Furthermore, a β-glucosidase from Gordonia terrae was shown to completely convert Re to Rg2 after 8 h, producing 1.47 mg/mL Rg2 from ginseng root extract [16]. These enzyme-based methods offer promising alternatives for the large-scale production of this pharmacologically active ginsenoside. Efficient preparation methods are the prerequisite for further pharmacokinetic and toxicological evaluation, which are currently insufficient.

Although basic physicochemical features of 20(S)-Rg2 and 20(R)-Rg2 have been addressed in the Introduction Section, systematic pharmacokinetic data describing absorption, distribution, metabolism and excretion for both epimers remain largely uncharacterized. Current sub-acute oral toxicological evidence from rodent studies suggests acceptable oral tolerance of Rg2. Still, data regarding long-term toxicity, genotoxicity, and reproductive toxicity remain absent, and safety observations obtained from animal work cannot be directly extrapolated to humans. These unaddressed knowledge gaps constitute key translational obstacles for further Rg2 research.

### 3.2. Pharmacokinetic Profile

Available pharmacokinetic data for purified ginsenoside Rg2 are limited to intravenous administration models. A validated HPLC method enabled simultaneous quantification of 20(R)-and 20(S)-Rg2 in rat plasma after tail-vein injection, revealing distinguishable elimination profiles between the two epimers. Following intravenous dosing, intact Rg2 was cleared predominantly via biliary–fecal routes: cumulative biliary excretion reached 27.2% within 5.5 h and fecal excretion 22.6% within 24 h, while urinary excretion of the parent compound was negligible [17]. In vitro incubation with rat liver microsomes identified several phase I metabolites of 20(S)-Rg2, some retaining SIRT1-activating bioactivity, suggesting that hepatic metabolism may contribute to bioactivation rather than simple detoxification [18].

For oral administration, protopanaxatriol-type ginsenosides undergo extensive deglycosylation in the gastrointestinal tract, and intact glycosylated saponins are poorly absorbed into systemic circulation [19,20]. Stepwise sugar hydrolysis generates more lipophilic aglycone metabolites, which serve as the predominant circulating bioactive forms after oral intake [21]. However, no published study has reported the oral absolute bioavailability of purified Rg2 epimers, and comprehensive ADME data—including intestinal absorption, tissue distribution, and metabolic clearance—remain unavailable, representing a major translational gap for interpreting in vitro findings.

### 3.3. Toxicological Safety Profile

Only one in vivo study has systematically evaluated the oral toxicity of purified Rg2 in rodents, reporting no acute lethality in mice at 10 g/kg and minimal sub-acute effects in a 28-day rat study (1.75–5 g/kg/day), with no mortality or significant organ-weight changes and only transient serum biochemical alterations at the highest dose [22]. In vitro, Rg2 did not induce obvious DNA damage in human keratinocytes and attenuated UV-B-triggered genotoxic stress, though these findings cannot replace in vivo genotoxicity assays. An EFSA opinion considered a ginseng crude extract tincture safe at proposed use levels in animals, but this conclusion applies to a multi-saponin mixture rather than purified Rg2 [23]. Overall, systematic toxicological data for the Rg2 monomer—particularly sub-chronic toxicity, genotoxicity, and reproductive–developmental toxicity—remain scarce, and extrapolation of current animal data to humans requires caution, as long-term safety cannot be reliably inferred from structural analogy alone [24].

## 4. Pharmacological Effects of Ginsenoside Rg2

### 4.1. Cardioprotective Effects of Rg2

**Figure 1:**
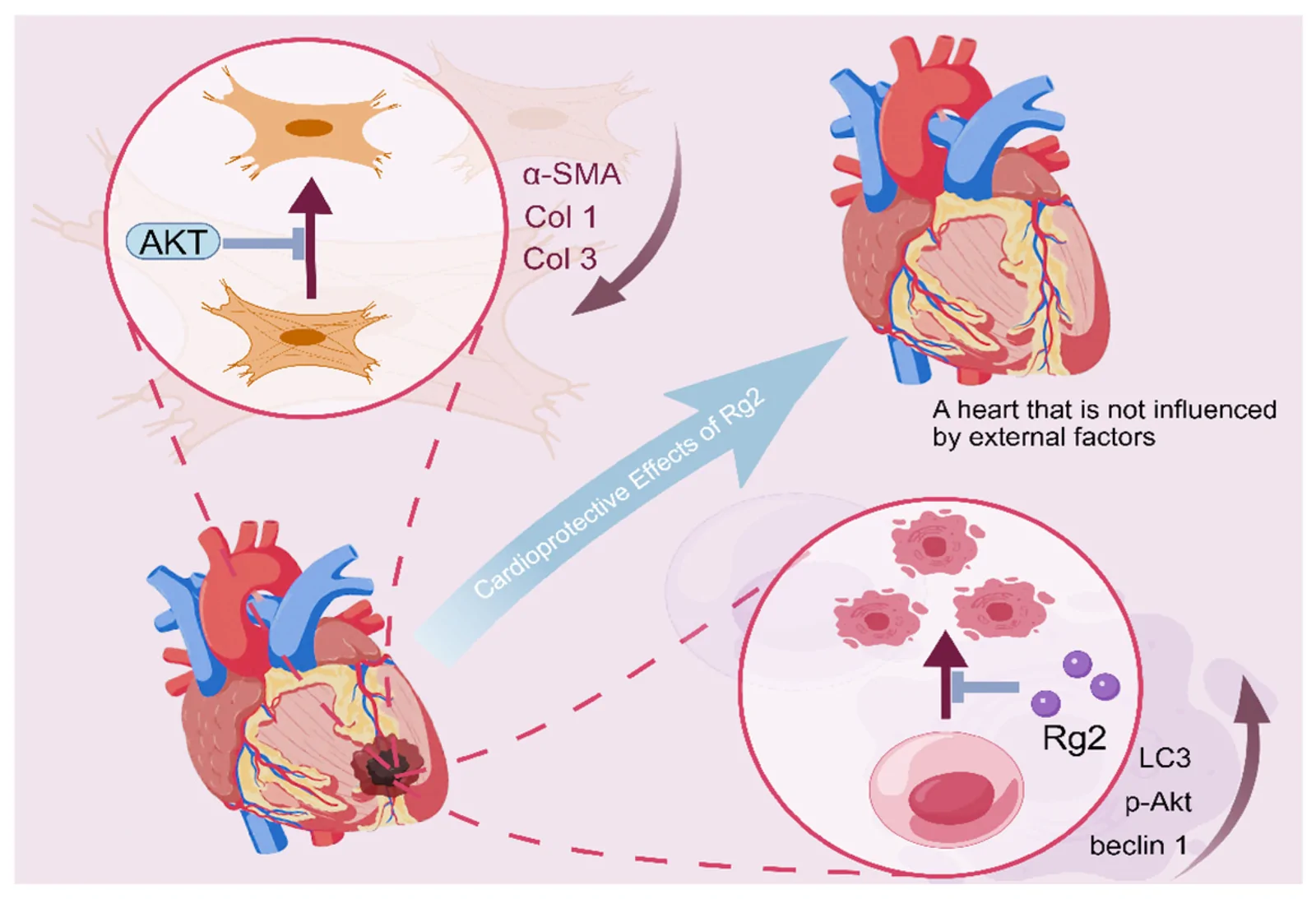
Cardioprotective mechanism of ginsenoside Rg2 via activating AKT signaling and regulating autophagy-related proteins. Ginsenoside Rg2 protects the heart by activating AKT signaling to suppress myocardial fibrosis and upregulating autophagy proteins to resist cardiotoxicity, which may improve cardiac function and prevent ventricular remodeling in pre-clinical models. Gradient purple arrows indicate the direction of regulation: upward arrows represent activation/upregulation, and downward arrows indicate inhibition/downregulation. Created with BioGDP [33] (https://biogdp.com).

Myocardial injury is one of the leading causes of death worldwide [25,26]. Although interventions such as cardiac stents have markedly reduced mortality rates [27], ventricular remodeling induced by myocardial infarction remains a pivotal clinical challenge that urgently needs to be addressed. It can lead to myocardial tissue fibrosis and structural remodeling of the heart, which in turn may trigger heart failure, malignant arrhythmias, and even endanger the patient’s life [28]. Pre-clinical in vivo studies suggest that Rg2 may activate the AKT signaling pathway in cardiac cells, downregulate fibrosis-related proteins, and mitigate myocardial fibrosis, which contributes to improved cardiac function in mice [29]. Wang et al. reported that Rg2 may improve the end-diastolic and end-systolic pressures of the heart, enhance cardiac diastolic and systolic function, and exert a significant dose-dependent effect in animal experiments [30]. In addition, pre-clinical findings indicate that Rg2 may protect cardiomyocytes from externally induced cardiotoxicity via upregulation of autophagy-related proteins [31]. Furthermore, Li et al. reported that Rg2 ameliorates myocardial ischemia/reperfusion (MI/R) injury by enhancing TAK1 phosphorylation, thereby preventing RIP1/RIP3 complex formation and inhibiting cardiomyocyte necroptosis in both H9c2 cells and a murine MI/R model [32]. The cardioprotective mechanism of Rg2 is shown in Figure 1.

### 4.2. The Regulatory Effects of Ginsenoside Rg2 on the Nervous System

#### 4.2.1. Improvement in Cognitive Function and Anti-Dementia Effects of Rg2

Alzheimer’s disease severely impairs patients’ cognitive functions and daily living abilities, placing a heavy burden on families and society [34,35]. Given the current shortage of effective pharmacotherapies for dementia [36], developing novel preventive and therapeutic strategies is urgently needed. Mechanistically, pre-clinical data suggest Rg2 may confer neuroprotection via coordinated dual-axis regulation: activation of the MAP-ERK cascade may attenuate hippocampal structural damage [37], while concurrent engagement of the PI3K/Akt survival signaling may directly block neuronal apoptosis [38,39]. In pre-clinical animal models, Rg2 has been reported to reduce β-amyloid and Tau burden and suppress pro-inflammatory factor expression [40]. Furthermore, proteomics analysis revealed that Rg2 improves scopolamine-induced memory impairment in mice by downregulating LGMN, LAMP1, and PSAP through the lysosomal pathway [41], which may collectively contribute to alleviating the brain inflammatory response. In summary, pre-clinical mouse studies suggest that Rg2 may improve cognitive function and mitigate pathological progression of Alzheimer’s-like pathology through synergistic actions across multiple targets and pathways.

#### 4.2.2. The Anxiolytic and Antidepressant Effects of Rg2

Prolonged anxiety is detrimental to both physical and mental health, disrupts cognitive functions, and can also reduce the quality of life [42]. When severe depression develops, it may be accompanied by lifelong illness and suicidal tendencies [43,44]. Pre-clinical studies suggest that Rg2 may produce antidepressant-like effects by modulating the hippocampal BDNF signaling pathway [45]. Additional rodent studies indicate that Rg2 may modulate post-traumatic stress-related phenotypes by altering progesterone, allopregnanolone, serotonin and their metabolite levels in rat brain and serum [46]. Collectively, these findings suggest that Rg2 may provide new ideas and research directions for the intervention of related neuropsychiatric disorders.

### 4.3. The Inhibitory Effect of Ginsenoside Rg2 on Inflammatory Response

#### 4.3.1. Hepatoprotective Effects of Rg2 Against Liver Injury

**Figure 2:**
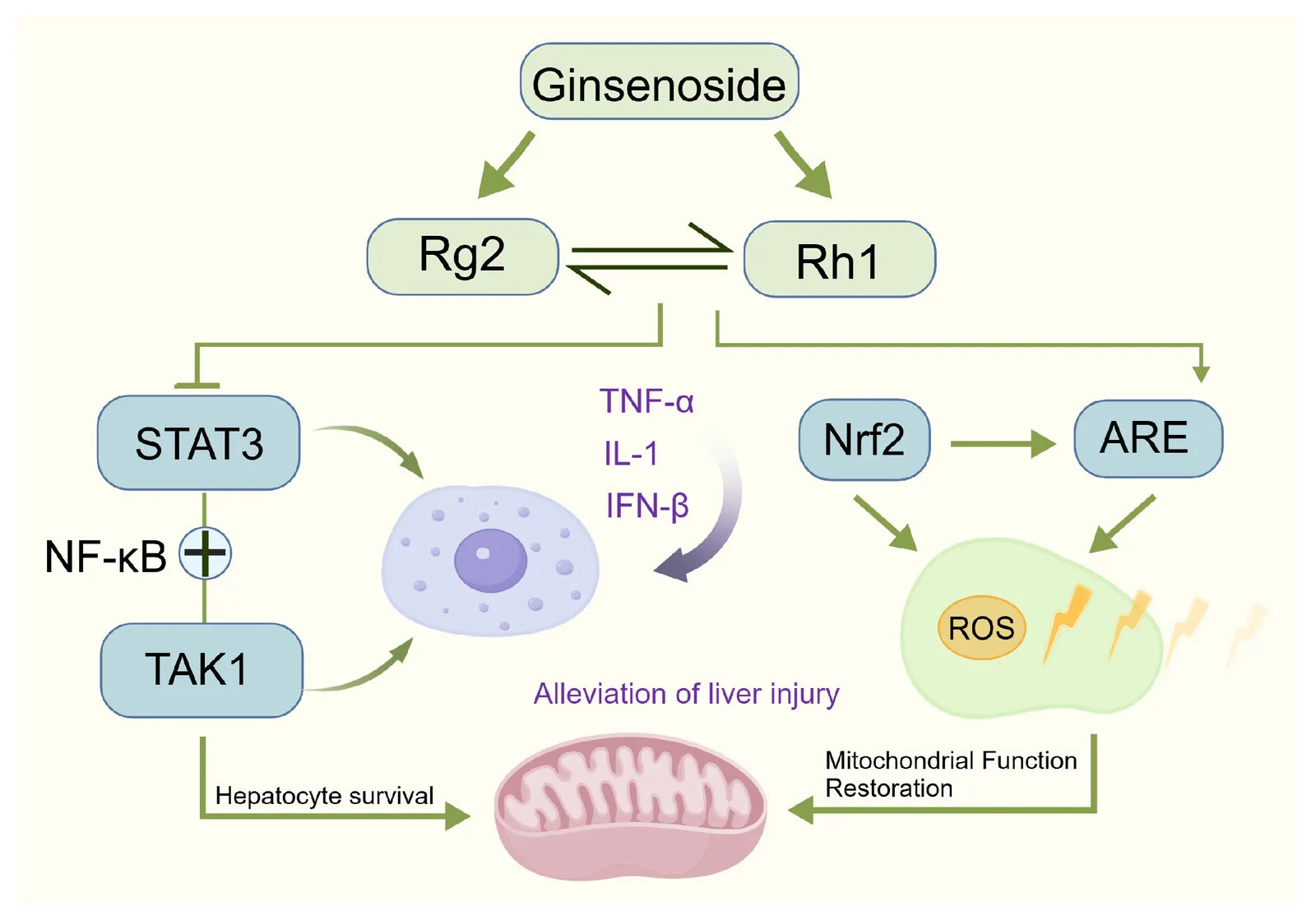
Ginsenoside Rg2 and its analogs via synergistic anti-inflammatory and antioxidant effects. Ginsenoside Rg2 and its analog Rh1 synergistically protect the liver by inhibiting the STAT3/TAK1/NF-κB inflammatory axis and activating the Nrf2/ARE antioxidant pathway, which reduces inflammation, eliminates ROS, restores mitochondrial function, and alleviates liver injury. Gradient purple arrows indicate inhibition and downregulation. Created with BioGDP (https://biogdp.com).

Liver inflammation is a common early manifestation of liver injury [47,48]. The incidence of liver injury is high and its onset is insidious, making it likely to lead to severe liver disease [49]. Its prevention and treatment remain a challenging issue that urgently needs to be addressed in clinical practice. Pre-clinical studies suggest that Rg2 may normalize dysregulated autophagy-related protein expression in the liver via modulation of the AKT/mTOR signaling pathway [50], which could ameliorate liver tissue injury and hepatic fibrosis in animal models. Rg2 can also regulate liver metabolism and enhance hepatocyte antioxidant capacity to maintain normal liver function [51,52]. Notably, Rg2 may function synergistically with its analog Rh1 to suppress the STAT3/TAK1 inflammatory axis [53], while potentiating Nrf2-driven antioxidant responses to scavenge excess reactive oxygen species and restore mitochondrial integrity [54]. This potential protective mechanism is illustrated in Figure 2.

#### 4.3.2. Anti-Inflammatory Effects of Rg2

Inflammation-related diseases are diverse, have a high incidence, and are difficult to treat [55], imposing a heavy burden on patients and clinical healthcare [56]. Pre-clinical mouse studies suggest that Rg2 may modulate the NF-κB/NLRP3 signaling pathway, which could improve blood glucose, blood-lipid profiles and renal function and slow the progression of diabetic nephropathy [57]. In atherosclerosis-related models, Rg2 may prevent IκBα degradation, suppress lipopolysaccharide-induced VCAM-1 and ICAM-1 expression, and reduce leukocyte–endothelial adhesion [58]. These observations point to potential vascular-protective activity against vascular inflammatory disorders. In ulcerative colitis models, studies have shown that Rg2 can regulate the NF-κB/NLRP3 pathway, inhibit inflammatory responses, and enhance intestinal-barrier integrity [59], thereby mitigating pathological manifestations of ulcerative colitis in mouse models. Collectively, these pre-clinical observations indicate the potential pharmacological activity of Rg2 across multiple inflammation-related disease models and offer directions for further investigation of multi-system inflammatory disorders.

### 4.4. The Anti-Tumor Effects of Ginsenoside Rg2

Malignant tumors are diseases that seriously threaten human health and even life, not only causing physical–mental suffering to patients [60] but also imposing a substantial economic burden [61]. Conducting safe and effective research on tumor prevention and control is of paramount importance for safeguarding human health. In breast-cancer models, Jeon et al. demonstrated that Rg2 downregulates anti-apoptotic proteins, suppresses ERK1/2 and Akt phosphorylation, induces G0/G1 cell-cycle arrest, and triggers AMPK-dependent mitochondrial dysfunction, collectively driving apoptotic cell death [62,63]. These in vitro findings indicate that Rg2 may trigger cancer-cell apoptosis and show potential against breast cancer; Kang et al. reported Rg2 may induce colorectal cancer-cell death partly via mitochondrial reactive-oxygen-species production [64]. These findings provide a new direction for the prevention and control of colorectal cancer. Note that these anti-tumor observations are mainly derived from in vitro tumor cell models; in vivo anti-tumor data remain limited and should be considered preliminary.

### 4.5. Antioxidant Effect of Ginsenoside Rg2

Oxidative stress is a core driving factor in the occurrence of many diseases [65,66]. Rg2 exhibits significant advantages in regulating oxidative stress and protecting against cellular damage. The antioxidant capacity of Rg2 has been validated across multiple cell types. In hepatocyte models, Rg2 may attenuate steatosis and oxidative injury by upregulating endogenous antioxidant enzymes [67]. In cardiomyocytes, it may suppress ROS paramount and modulate Bcl-2 family proteins to counteract H_2_O_2_-triggered apoptosis [68]. In hippocampal neurons, its protective potential may involve mitigation of hypoxia-induced Ca^2+^ overload and free-radical scavenging [69]. Collectively, these findings point to its protective potential against hypoxia-related hippocampal neuronal damage.

### 4.6. Anti-Aging-Related Effects of Ginsenoside Rg2

Aging is a natural process in the development of life [70]. However, through scientific intervention and precise regulation, the risk of developing aging-related diseases can be reduced [71]. In mouse brain-aging models, Rg2 may ameliorate memory deficits and cholinergic dysfunction by downregulating aberrant overexpression of aging-associated proteins [72]. Accumulating data suggest AMPK-dependent autophagy may represent one important contributing mechanism across multiple tissues: beyond the brain, Rg2 may reverse stem-cell senescence and mitigate aging-associated kidney injury through maintenance of mitochondrial homeostasis [73,74,75]. Mitochondrial autophagy and homeostatic regulation are hypothesized to underlie many of these phenotypes [76]. These pre-clinical results provide experimental clues for further research on anti-aging interventions.

### 4.7. Miscellaneous Biological Functions of Ginsenoside Rg2

**Figure 3:**
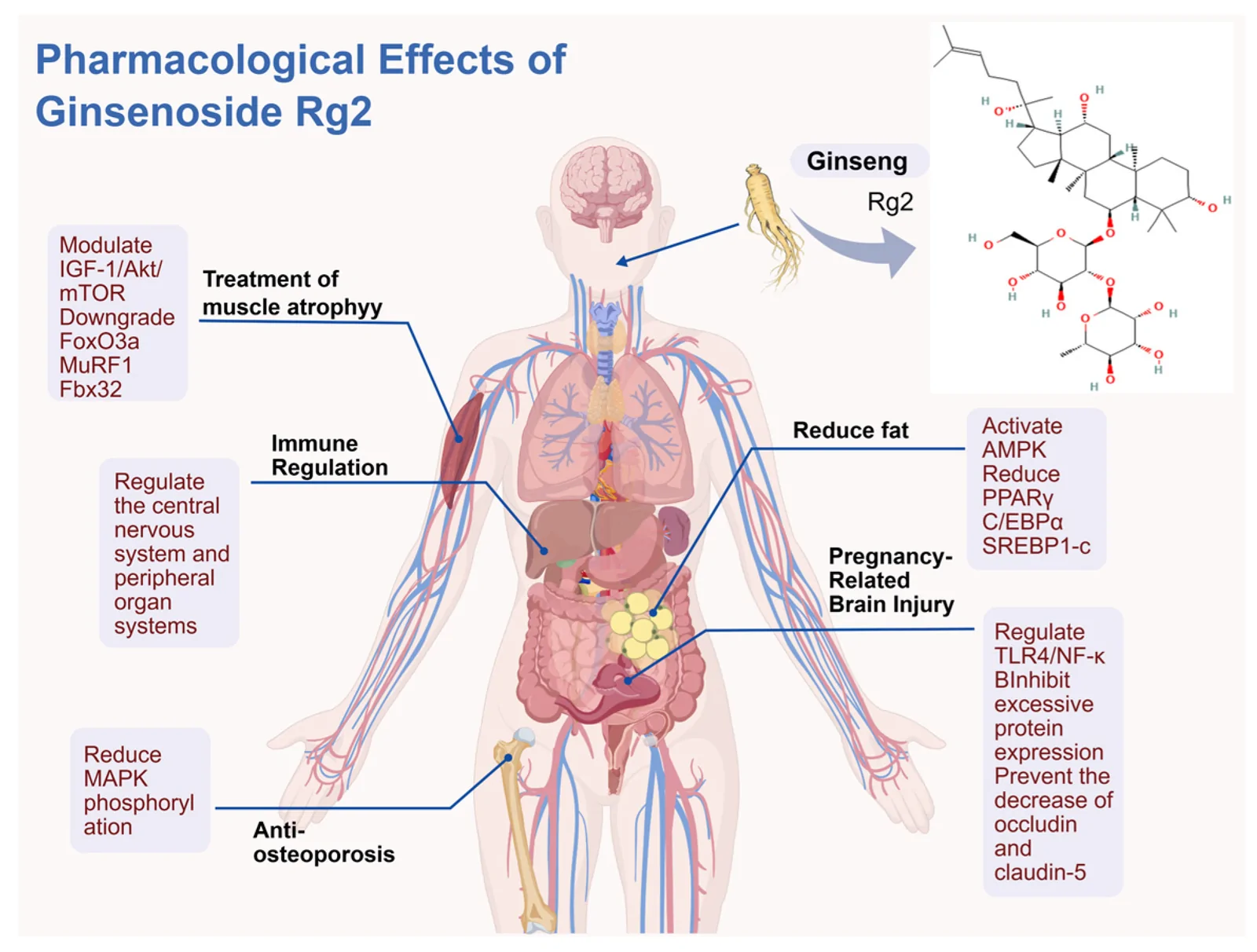
Multifunctional biological activities of ginsenoside Rg2 in neuroprotection, anti-osteoporosis, anti-muscle atrophy, metabolic regulation, and immunomodulation. Ginsenoside Rg2 exerts multi-system pharmacological effects via multiple signaling pathways: it protects against pregnancy-related brain injury via TLR4/NF-κB, inhibits osteoporosis via MAPK, alleviates muscle atrophy via IGF-1/Akt/mTOR and SIRT1/PGC-1α, and regulates metabolism via AMPK, with synergistic anti-inflammatory activity and good safety. Created with BioGDP (https://biogdp.com).

In addition to the aforementioned pharmacological activities (e.g., cardioprotection, neuroprotection, anti-inflammatory, anti-tumor, antioxidant, and anti-aging effects), Rg2 also exhibits promising effects in reproductive, musculoskeletal, metabolic, and immune systems. For instance, in preeclampsia-associated intracerebral hemorrhage rodent models, Rg2 may preserve blood–brain barrier integrity via TLR4/NF-κB modulation [77]. In the musculoskeletal system, Rg2 may suppress osteoclast differentiation under bone-homeostasis experimental conditions [78], and it may counteract dexamethasone-induced muscle atrophy through IGF-1/Akt/mTOR activation [79]. Regarding metabolic regulation, its AMPK-driven anti-adipogenic effect suggests potential for obesity-related research [80]. Finally, in the immune system, Rg2 may exert bidirectional immunomodulatory activity and produce synergistic anti-inflammatory efficacy when combined with Rg5, Rk1, or Rh1 [81], although the epimer compositions of these combinations were not specified in the original study. These mechanisms are summarized in Figure 3. A comprehensive summary of the experimental models, doses, treatment durations, and outcome measures from the 32 primary studies is presented in Table 2 and Table 3.

**Table 2:** Summary of the cardioprotective, neuroprotective, and anti-aging effects of ginsenoside Rg2 from original studies.

Pharmacological Effect	Model (Pathology)	Epimer	Dose & Route	Time	Outcome (Direction of Effect)	Positive Control	Evidence Type	Ref.
Anti-myocardial fibrosis	ICR mice, MI-induced	N/A	5/10/20 mg/kg i.g.	4 weeks	Fibrotic area ↓; p-AKT ↑; Col-1/Col-3 ↓; improved cardiac function (dose-dependent)	Not reported	in vivo	[29]
Anti-myocardial fibrosis	Wistar rats, isoproterenol-induced myocardial fibrosis	N/A	5/20 mg/kg i.g.	28 d	LVEDP/LVESP improvement; TGF-β1/Smad3 ↓; collagen deposition ↓	Not reported	in vivo	[30]
Anti-cardiotoxicity	H9c2 human cardiomyocytes, trastuzumab-induced injury	N/A	20–200 µM	24 h	Cell viability ↑; LC3-II/beclin-1/p-Akt ↑; apoptosis ↓	Not reported	in vitro	[31]
Anti-myocardial I/R injury (anti-necroptosis)	ICR mice, C57BL/6 mice, C57BL/6 mice, MI/R; H9c2 cells, H/R	N/A	50 mg/kg i.v.; H/R model (in vitro)	MI/R mouse model; H9c2 H/R model	Cardiomyocyte death ↓; p-RIP1/p-RIP3/p-MLKL ↓; RIP1–RIP3 necrosome formation ↓; p-TAK1 ↑; myocardial injury area ↓	Not reported	in vivo + in vitro	[32]
Neuroprotection/anti-AD	3xTg-AD mice	N/A	198.4 mg/kg/day i.g.	6 weeks	MAPK-ERK ↑; hippocampal vascular-neuronal unit damage ↓	Not reported	in vivo	[37]
Anti-neuronal apoptosis	PC12 cells, Aβ_25–35_ injury	N/A	10–40 µM	24 h	PI3K/Akt ↑; Bax ↓/Bcl-2 ↑; neuronal apoptosis ↓	Not reported	in vitro	[38]
Memory-protective/anti-AD	SD rats, Aβ_25–35_-induced memory impairment	N/A	10/20 mg/kg i.g.	21 d	Learning-memory performance ↑; neuronal apoptosis ↓	Not reported	in vivo	[39]
Memory improvement	ICR mice, scopolamine-induced memory deficit	N/A	20 mg/kg i.g.	30 d	Aβ/p-Tau ↓; pro-inflammatory cytokines ↓	Not reported	in vivo	[40]
Antidepressant-like activity	C57BL/6J mice, chronic mild stress (CMS) model	N/A	10/20 mg/kg i.g.	6 weeks; (CMS); treatment in last 2 weeks	FST/TST immobility ↓; sucrose preference ↑; hippocampal BDNF-TrkB-CREB signaling ↑; TrkB shRNA blocked the effect	Fluoxetine, 20 mg/kg	in vivo	[45]
Anxiolytic/PTSD-like intervention	SD rats, SPS PTSD model	N/A	5/10/20 mg/kg i.g.	14 d	Anxiety-related behaviors ↓; progesterone/allopregnanolone/5-HT metabolite modulation	Sertraline, 15 mg/kg i.g.	in vivo	[46]
Anti-hypoxic neuronal protection	Primary rat hippocampal neurons, hypoxia challenge	N/A	10–1000 nM	12 h	Neuronal survival ↑; Ca^2+^ overload ↓; free-radical scavenging	Not reported	in vitro	[69]
Anti-brain aging	ICR mice, D-galactose-induced brain aging	N/A [Co-tested:Rg2/PPT/AFG]	5/10/20 mg/kg i.g.	8 weeks	p53/p21/p16ink4α ↓; mitophagy ↑; memory impairment ameliorated	Not reported	in vivo	[72]
Stem-cell senescence protection	Porcine mesenchymal stem cells	N/A [Co-tested:Rg2/PPT/AFG]	10–40 µM	3 d	Cell proliferation & stemness maintenance ↑; AMPK-autophagy activation	Not reported	in vitro	[73]
Anti-kidney aging	SAMP8 natural aging mice	N/A [Co-tested:Rg2/PPT/AFG]	20 mg/kg i.g.	8 weeks	Age-related kidney pathological injury ↓; mitochondrial homeostasis improvement	Not reported	in vivo	[74]

Abbreviations: AD, Alzheimer’s disease; Col-1/3, collagen type I/III; CUMS, chronic unpredictable mild stress; D-gal, D-galactose; i.g., intragastric administration; i.p., intraperitoneal injection; LAMP1, lysosomal-associated membrane protein 1; LGMN, legumain; LVEDP, left ventricular end-diastolic pressure; LVESP, left ventricular end-systolic pressure; MI, myocardial infarction; MSCs, mesenchymal stem cells; N/A, not available (epimer not specified in the original study); PSAP, prosaposin; PTSD, post-traumatic stress disorder; SPS, single prolonged stress; MI/R, myocardial ischemia/reperfusion. Symbols: ↑, increase; ↓, decrease. Reference numbers correspond to the reference list in the main text. All data were extracted from original research articles cited in the main text. Review articles were not included in this table. Epimer annotation: Rg2/PPT/AFG indicates that the original study compared ginsenoside Rg2 with 20(S)-protopanaxatriol (PPT) and American ginseng fraction (AFG) under the same experimental conditions.

**Table 3:** Summary of the anti-inflammatory, anti-tumor, antioxidant, and other pharmacological effects of ginsenoside Rg2 from original studies.

Pharmacological Effect	Model (Pathology)	Epimer	Dose & Route	Time	Outcome (Direction of Effect)	Positive Control	Evidence Type	Ref.
Hepato-protective/anti-fibrosis	C57BL/6 mice, CDAHFD-induced hepatic fibrosis	N/A	20 mg/kg i.g.	8 weeks	AKT/mTOR pathway modulation; autophagy normalization; liver fibrosis ↓	Not reported	in vivo	[50]
Anti-aging-related liver injury	SAMP8 aged mice	N/A	10–40 mg/kg i.g.	4 weeks	Caspase-8-mediated pyroptosis ↓; gut-microbiota remodeling	Not reported	in vivo	[51]
Anti-NASH/metabolic hepatitis	MCD-diet-fed mice & HepG2 cells	N/A [Co-tested:Rg2 + Rh1]	25–100 µM (cell); 10–40 mg/kg i.g. (in vivo)	24 h/8 weeks	Nrf2 antioxidant activation; inflammation/fibrosis ↓	Not reported	both	[52]
Synergistic liver protection	Rg2 + Rh1, LPS-stimulated HepG2 cells	N/A [Co-tested:Rg2 + Rh1]	25–100 µM	Not specified	STAT3/TAK1 inflammatory axis ↓; Nrf2-ARE ↑; ROS ↓	Not reported	in vitro	[53]
Anti-metabolic disorder	C57BL/6 mice, HFD-induced metabolic disease	N/A	20 mg/kg i.p.	8 weeks	SIRT1 ↑; lipid-glycometabolic improvement; hepatic steatosis ↓	Not reported	in vivo	[54]
Anti-diabetic nephropathy	db/db mice, HFD/STZ-induced diabetic nephropathy	N/A	10–40 mg/kg i.g.	8 weeks	NF-κB/NLRP3 pyroptosis pathway ↓; blood-glucose/renal-function improvement	Not reported	in vivo	[57]
Anti-vascular inflammatory (atherosclerosis-relevant)	HUVECs, LPS-stimulated endothelial injury	N/A	1–50 µM	1–8 h	IκBα-degradation ↓; VCAM-1/ICAM-1 ↓; leukocyte-endothelial adhesion ↓	Not reported	in vitro	[58]
Anti-colitis/intestinal barrier protection	C57BL/6 mice, DSS-induced colitis	N/A	10/20 mg/kg i.g.	7–10 d	NF-κB/NLRP3 ↓; pro-inflammatory factors ↓; intestinal-barrier integrity ↑	5-ASA	in vivo + in vitro	[59]
Anti-breast-cancer cell activity	MCF-7 human breast-cancer cells	20(S)-Rg2	25–100 µM	48 h	ROS/AMPK ↑; G0/G1 cell-cycle arrest; mitochondrial dysfunction; apoptosis ↑	Not reported	in vitro	[62]
Anti-breast-cancer cell activity	MCF-7 human breast-cancer cells	20(S)-Rg2	25–100 µM	48 h	ROS-dependent mitochondrial-oxidative injury; cancer-cell apoptosis ↑	Not reported	in vitro	[63]
Anti-colorectal-cancer cell activity	HT-29, HCT116 colon-cancer cells	N/A	100–400 µg/mL	48 h	Mitochondrial-ROS generation; autophagy–apoptosis-mediated cancer-cell death	Not reported	in vitro	[64]
Cardiomyocyte anti-oxidative protection	H9c2 cardiomyocytes, H_2_O_2_ oxidative damage	N/A	25–100 µM	4 h	ROS generation ↓; Bcl-2-family modulation; cell viability ↑; LDH/MDA ↓	Not reported	in vitro	[68]
Hippocampal-neuron anti-hypoxia/anti-oxidation	Primary hippocampal neurons, hypoxia-induced injury	N/A	10–1000 nM	12 h	Ca^2+^-overload mitigation; free-radical scavenging; neuronal survival ↑	Not reported	in vitro	[69]
Preeclampsia-related intracerebral-hemorrhage brain protection	SD rats, PE + ICH model	N/A	10–40 mg/kg i.v.	3 d	TLR4/NF-κB ↓; blood–brain-barrier preservation; brain edema ↓	Not reported	in vivo	[77]
Anti-osteoclast-differentiation/anti-osteoporosis	BMMs, RANKL-stimulated osteoclastogenesis	N/A	10–40 µM	7 d	MAPK-NFATc1 signaling ↓; osteoclast-formation inhibition	Not reported	in vitro	[78]
Anti-dexamethasone-induced muscle atrophy	C2C12 myotubes, dexamethasone-atrophy model	N/A [Co-tested:Rg2 + Rh1 + Rg3]	10–50 µM	48 h	IGF-1/Akt/mTOR & PGC-1α-mitochondrial biogenesis ↑; myotube diameter ↑	Not reported	in vitro	[79]
Anti-adipogenic/lifespan-regulating effect	C. Elegans model	N/A	50–200 µM	Lifelong treatment	Lipid-metabolism-modulation; stress-response-pathway activation; lifespan extension phenotype	Not reported	in vivo	[80]

Abbreviations: BMMs, bone marrow-derived macrophages; CDAHFD, choline-deficient, high-fat diet; DSS, dextran sulfate sodium; HFD, high-fat diet; HUVECs, human umbilical vein endothelial cells; i.g., intragastric administration; i.p., intraperitoneal injection; i.v., intravenous injection; MCD, methionine-choline deficient; N/A, not available (epimer not specified in the original study); PE + ICH, preeclampsia-associated intracerebral hemorrhage; RANKL, receptor activator of nuclear factor-κB ligand; STZ, streptozotocin; 5-ASA, 5-aminosalicylic acid (mesalazine). Symbols: ↑, increase; ↓, decrease. Reference numbers correspond to the reference list in the main text. All data were extracted from original research articles cited in the main text. Review articles were not included in this table. Epimer annotation: Co-tested:Rg2 + Rh1 indicates that the study tested a ginsenoside mixture containing both Rg2 and Rh1 without individual epimer specification for Rg2.

### 4.8. Limitations of the Current Evidence Base

It should be noted that evidence for ginsenoside Rg2 pharmacological activities mainly comes from pre-clinical studies and should be regarded as preliminary. Briefly, several biological effects are supported by single studies, and most studies do not specify which 20(S)- or 20(R)-Rg2 epimer was used, limiting cross-study comparison. These limitations are discussed in detail in Section 5.

## 5. Conclusions and Outlook

Ginsenoside Rg2 is a secondary saponin derived from the hydrolysis of ginsenoside Re. With low toxicity, abundant natural sources, and multi-target, multi-pathway pharmacological properties, Rg2 has attracted widespread academic attention and become a popular research focus. Current studies have demonstrated that Rg2 exhibits anti-inflammatory, antioxidant, anti-tumor, neuroprotective, and anti-aging activities, although its precise molecular mechanisms remain incompletely clarified. In the field of cardiovascular protection, Rg2 can exert multiple effects of myocardial protection, cardiac function amelioration and arrhythmia suppression after myocardial infarction through key pathways such as AKT and TGF-β1/Smad; in terms of central nervous system regulation, Rg2 can not only mitigate nerve injuries such as cerebral hemorrhage and cerebral ischemia and reperfusion. It can also enhance cognitive function, antagonize dementia, and also have the effect of anti-anxiety and anti-depression neuropsychial protection. In the field of anti-inflammatory effects, the effect of Rg2 covers multi-system inflammatory diseases such as liver injury and diabetic nephropathy, and the core is to regulate classic inflammatory pathways such as NF-κB and NLRP3. At the same time, it also has clear anti-tumor, antioxidant and anti-aging activities. The core mechanism revolves around ROS-mediated cell regulation, key signal pathway activation and oxidative stress suppression. The above-mentioned diverse pharmacological profiles and suggested molecular mechanisms provide a preliminary experimental basis that may inform future translational research on Rg2, though further validation in clinical settings is required.

Nevertheless, several critical limitations still hinder the current understanding of Rg2. Most pharmacological evidence originates from in vitro cell assays and pre-clinical animal models, while only a limited number of human-relevant studies are available and the clinical data remain insufficient. Notably, most previous studies did not specify which 20(S)- or 20(R)-Rg2 epimer was used, resulting in ambiguous and incomparable pharmacological data, as reflected in the predominantly “N/A” entries in Table 2 and Table 3. In addition, many biological effects are supported only by individual studies and require further validation. Furthermore, potential publication bias exists, as negative or inconsistent results are rarely reported. Collectively, these limitations indicate that the current evidence is insufficient to confirm the clinical efficacy of Rg2, and the conclusions of this review should be interpreted with caution.

Future investigations should prioritize large-scale, well-designed clinical studies to clarify the optimal dosage, administration route, and safety profile of Rg2 in humans. In-depth mechanistic exploration is also required to elucidate the crosstalk among different signaling pathways and construct a more systematic regulatory network. Moreover, it is necessary to optimize existing extraction and preparation techniques to improve the purity and yield of Rg2, as well as develop novel nano-formulations and targeted delivery systems to overcome its low bioavailability. Further exploration of combination therapy strategies and potential therapeutic effects in more disease models is also warranted. With continuous and standardized investigation, the field may eventually progress toward clinical evaluation, potentially offering a novel natural therapeutic option for multiple diseases and providing new insights for the development of active phytochemical components.

## Figures and Tables

**Table 1:** Chemical structures of 20(S)-ginsenoside Rg2 and 20(R)-ginsenoside Rg2.

Chemical Name	Molecular Formula	Molecular Weight	Chemical Structure	C-20 Stereocenter
20(S)-ginsenoside Rg2	C_42_H_72_O_13_	785.0 g/mol	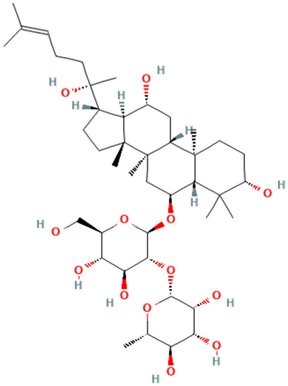	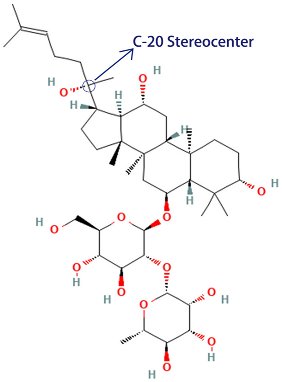
20(R)-ginsenoside Rg2	C_42_H_72_O_13_	785.0 g/mol	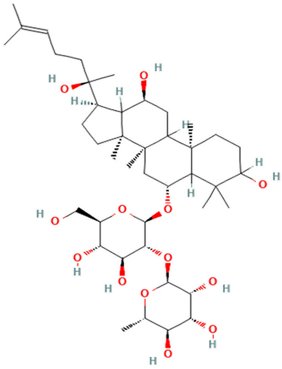	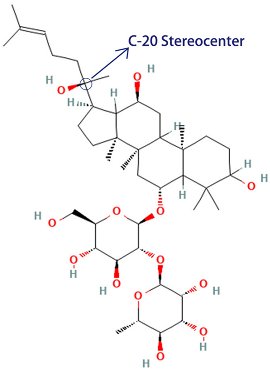

Chemical structures were obtained from PubChem (https://pubchem.ncbi.nlm.nih.gov/) and are used under the public domain license. Table layout is original.

## Data Availability

No new data were created or analyzed in this study. Data sharing is not applicable to this article.
